# Adenosine and adenosine receptors in metabolic imbalance-related neurological issues

**DOI:** 10.1016/j.biopha.2024.116996

**Published:** 2024-06-18

**Authors:** Mi-Hyeon Jang, Juhyun Song

**Affiliations:** a Department of Neurosurgery, Robert Wood Johnson Medical School, Rutgers, The State University of New Jersey, Piscataway, NJ 08854, United States; b Department of Anatomy, Chonnam National University Medical School, Hwasun 58128, Republic of Korea

**Keywords:** Adenosine, Adenosine receptors, Metabolic imbalance, Blood-brain barrier (BBB), Insulin resistance

## Abstract

Metabolic syndromes (e.g., obesity) are characterized by insulin resistance, chronic inflammation, impaired glucose metabolism, and dyslipidemia. Recently, patients with metabolic syndromes have experienced not only metabolic problems but also neuropathological issues, including cognitive impairment. Several studies have reported blood-brain barrier (BBB) disruption and insulin resistance in the brain of patients with obesity and diabetes. Adenosine, a purine nucleoside, is known to regulate various cellular responses (e.g., the neuroinflammatory response) by binding with adenosine receptors in the central nervous system (CNS). Adenosine has four known receptors: A1R, A2AR, A2BR, and A3R. These receptors play distinct roles in various physiological and pathological processes in the brain, including endothelial cell homeostasis, insulin sensitivity, microglial activation, lipid metabolism, immune cell infiltration, and synaptic plasticity. Here, we review the recent findings on the role of adenosine receptor-mediated signaling in neuropathological issues related to metabolic imbalance. We highlight the importance of adenosine signaling in the development of therapeutic solutions for neuropathological issues in patients with metabolic syndromes.

## 1. Introduction

Metabolic syndromes such as obesity and diabetes affect approximately 45 % of the global population, with their incidence steadily increasing worldwide [1,2]. These syndromes are characterized by insulin resistance, hyperglycemia, and hyperlipidemia [3]. Recently, metabolic syndromes have been reported to include some neuropathological issues such as blood-brain barrier (BBB) disruption, neurovascular dysfunction, neuroinflammation, cognitive dysfunction, brain insulin resistance including systemic insulin resistance, and hyperlipidemia [4–8]. It is crucial to address these neuropathological complications in individuals with metabolic syndromes given their increasing prevalence on a global scale. Therefore, further research is necessary to develop effective treatments for these conditions. Adenosine is a purine nucleoside that acts as a neurotransmitter and neuromodulator in the central nervous system (CNS). Adenosine is a major component in energy production and can be produced during the catabolism of adenosine triphosphate (ATP) [9]. Four adenosine receptors, i.e., A1R, A2AR, A2BR, and A3R, are G protein-coupled receptors extensively distributed throughout the brain. Notably, the inhibitory adenosine A1 receptor (A1R) and the facilitatory adenosine A2A receptor (A2AR) are pre-dominantly found in various brain regions, including the amygdala, hippocampal formation, hypothalamus, and cerebral cortex [10–12]. The four adenosine receptors show different affinities for adenosine [11]. For example, A1R is known to have the highest affinity of adenosine at approximately 70 nM, and whereas A2AR is known to have a lower affinity at approximately 150 nM [11]. A1R and A2AR are responsible for mediating presynaptic and postsynaptic neurotransmitter modulation [13,14]. A1R and A2AR are present in the neurons and glia and can regulate brain cellular homeostasis [9,10]. Furthermore, A1R and A2AR play a role in the modulation of synaptic transmission and formation of neuronal circuits [15,16], as well as various functions such as mood and motor control [17]. Adenosine receptors can also influence dopamine release indirectly by modulating the activity of other neurotransmitter systems, such as glutamate and GABA, which in turn affect dopaminergic signaling [18,19]. Furthermore, adenosine receptors regulate the release of neurotransmitters by modulating calcium entry and sensitivity at nerve terminals [18,19]. Previous studies reported that A1R activation promotes the internalization of GluA1 and GluA2-containing α-amino-3--hydroxy-5-methyl-4-isoxazolepropionic acid (AMPA) receptors [20,21]. A2AR activation is involved in the modulation of hippocampal long-term potentiation (LTP) in learning and memory [22]. In addition, A2AR-mediated signaling promotes the regulation of cytokine secretion and inflammatory signal transduction in microglia under inflammatory conditions in the brain [23–25]. A2AR is found in brain endothelial cells and controls brain neurovascular homeostasis [26]. A1R and A2AR are also present in astrocytes [16,27]. A1R mediates immune responses, whereas A2AR mediates astrocyte reactivity [28,29], induces gliosis [29], promotes glutamate release and synaptic transmission [30], and regulates memory consolidation [31]. Extracellular and intracellular adenosine is involved in several physiological functions including inflammation, immune responses, energy metabolism, neuronal function, DNA methylation, and BBB permeability in the CNS [32,33]. Dysregulation of adenosine homeostasis can result in various pathophysiological issues, such as memory loss, sleep disorders, and neuroinflammation [19,34]. In the brain, the adenosine modulation system fine-tunes glutamatergic synapses through A1R and A2AR, thereby contributing to the quality of the information encoded in neural networks [19]. Extracellular and intracellular adenosine is modulated by different enzymes such as adenosine kinase (ADK), ectonucleotidases, endonucleotidases, and adenosine deaminase (ADA) [35]. The secretion of adenosine from intracellular sources through transporters and the extracellular conversion of secreted adenine nucleotides by CD39 (ectonucleoside triphosphate diphosphohydrolase) and CD73 (ecto-5′-nucleotidase) are two modes of extracellular adenosine generation [36]. In the CNS, neurons release a high concentration of ATP, which can be converted to adenosine through CD39 and CD73 in neuronal diseases such as seizure [37]. Adenosine homeostasis is important for cellular metabolism regulation, synaptic function, and glia-neuron interactions involving the activation of AMP-activated protein kinase (AMPK) as a key energy sensor [16,38–41]. Some studies demonstrated the direct role of adenosine receptors in neuronal and astrocytic metabolism based on the pyruvate carboxylase/pyruvate dehydrogenase ratio [42,43]. A study reported the regulation of A1R in brain mitochondrial pathways including mitochondrial metabolism and during the reoxygenation process under hypoxia in the hippocampus [44]. A recent study indicated that adenosine receptors, as putative targets of caffeine, could contribute to synaptic transmission and neuronal circuits by modulating brain metabolic activity after regular caffeine intake [45]. The activation of adenosine receptors caused by chronic moderate caffeine intake at a moderate dose could modulate synaptic metabolism [46]. Moreover, A2AR could mediate the ergogenic effects of caffeine and may be involved in brain metabolic activity [47]. Duarte et al. showed that caffeine consumption could prevent synaptotoxicity, memory deficit, and metabolic imbalance in a diabetic animal model [48–50] and a hypoxia injury model [44] by regulating the activation of adenosine receptors. Additionally, A2AR blockade could inhibit hippocampal neuronal cell death and apoptotic-like synaptotoxicity in the hippocampus with mitochondria as the central hub of metabolism [51]. Adenosine receptor ligands prevent necrotic cell death in cerebellar granule neurons, which are involved in mitochondrial function [52]. Several studies indicated that adenosine is involved in various neuropathological processes including synaptic plasticity [53], astrocytic activity [16], cognitive function [54], motor function [55], sleep pattern [56], pain [57], ischemic stroke [58], traumatic brain injuries (TBI) [59], Parkinson’s disease (PD) [60], epilepsy [61], amyotrophic lateral sclerosis (ALS) [62], and Alzheimer’s disease (AD) [63]. Recent studies have attempted to treat neurological issues through A2AR blockade using antagonists [62,64–66]. Based on previous findings, we believe that adenosine receptor blockade could represent a promising therapeutic strategy for addressing neurological issues associated with metabolic imbalances. Here, we review the recent findings on the role of adenosine receptor-mediated signaling in insulin resistance, dyslipidemia, BBB disruption, and cognitive impairment in the brain.

## 2. Adenosine signaling, insulin resistance, and lipid metabolism

The signaling pathways mediated by adenosine-A2AR and A2BR are known to regulate lipolysis, glucose metabolism, and insulin sensitivity in the adipose tissues and skeletal muscles of patients with metabolic syndromes [67–69]. ATP serves as a danger signal in the brain, affecting the functions of astrocytes and other glial cells [16]. The primary source of adenosine is the activity of specific enzymes such as CD39, which converts ATP into AMP, and CD73, which dephosphorylates AMP to produce adenosine [70]. Adenosine, a metabolic product of ATP, can be recycled to reconstitute ATP and functions through four adenosine receptors: A1, A2A, A2B, and A3 [71]. The activation of A1R leads to a reduction in cAMP production, subsequently inducing lipolysis through cAMP-dependent protein kinase and lipases [72]. Studies have demonstrated that adenosine can improve insulin sensitivity, reduce blood glucose levels [73–75], stimulate glucagon secretion [76], and increase hepatic glycogenolysis [77]. In adipose tissues, adenosine can suppress lipolysis and promotes lipogenesis via A1R signaling [67,78]. Adenosine signaling through A1R has been observed to enhance insulin sensitivity and glucose tolerance, particularly under a high-fat Western diet [67, 79]. Additionally, A2BR signaling has been implicated in insulin resistance and the reduction of glucose levels [80]. Treatment with an A1R agonist has been shown to improve insulin sensitivity and decrease free fatty acid levels in obese rats [81]. Furthermore, deficiency in both A1R and A2BR has been associated with increased leptin levels, affecting body weight regulation and satiety, as well as elevated fasting glucose and insulin levels in the blood of high-fat diet mice [79,82]. A study has demonstrated that adenosine produced by adipocytes can stimulate the secretion of leptin in a phospholipase C-protein kinase C (PLC-PKC)-dependent manner [83]. Additionally, A2BR-mediated signaling can modulate lipid synthesis in the liver and the levels of cholesterol and triglycerides in the blood [84] through the activation of cAMP-dependent protein kinase (PKA) [78,85]. Other studies reported that adenosine signaling via A1R can ameliorate insulin resistance and reduce plasma levels of triglycerides, glycerol, and free fatty acids [72, 78,86]. Exogenous adenosine signaling through A2AR enhances gluconeogenesis [87]. A2AR-mediated signaling also promotes reverse cholesterol transport [88], which is known for its anti-diabetic potential [74,89]. In an in vitro study using rat hepatocytes, A1R activation could enhance glycogenolysis, whereas A2AR activation could increase gluconeogenesis [77]. Another study showed that A2BR activation could induce both glyconeogenesis and glycogenolysis [90]. In the process of cholesterol efflux, adenosine 5-triphosphate-binding cassette transporter A1 (ABCA1) and G1 (ABCG1) interact with apolipoprotein AI (ApoA1) and high-density lipoprotein (HDL), respectively, facilitating the transport of cholesterol content from macrophages to blood-circulating lipoproteins [91]. A2AR inhibition with the antagonist ZM241385 may lead to high lipid accumulation in macrophages and endothelial cells, promoting cell formation by suppressing the activation of proteins responsible for cholesterol efflux, such as ABCA1, ABCG1, and CYP27A1 [92]. Adenosine and adenosine receptors play a role in modulating cholesterol content in macrophages by enhancing cholesterol efflux through A2AR activation and suppressing inflammation through A2AR and A2BR activation, which are implicated in the development of atherosclerosis and dyslipidemia [93]. Previous studies have observed imbalanced glucose metabolism and impaired lipid profiles, including high total cholesterol, high low-density lipoprotein (LDL), and high triglyceride levels in A2BR knockout mice [82,94]. A study demonstrated that A2BR knockout macrophages could induce dyslipidemia, and restoring A2BR expression could have a positive impact on abnormal lipid profiles [95]. Following A2BR restoration in macrophages, improvements in body weight, insulin sensitivity, abnormal lipid profiles, and dysregulated adipokine levels were observed [94]. In A2AR/ApoE double-knockout mice, total cholesterol and LDL cholesterol levels were found to increase by 30 % in the plasma [96]. In addition, a study demonstrated that A1R knockout in mice could increase the probability of developing atherosclerosis [97].

Overall, the findings suggest that adenosine and adenosine receptors play a critical role in regulating energy metabolism, insulin sensitivity, glucose metabolism, and lipid profiles under metabolic imbalance conditions.

## 3. Adenosine signaling, neuroinflammation, and immune system

**Fig. 1.**
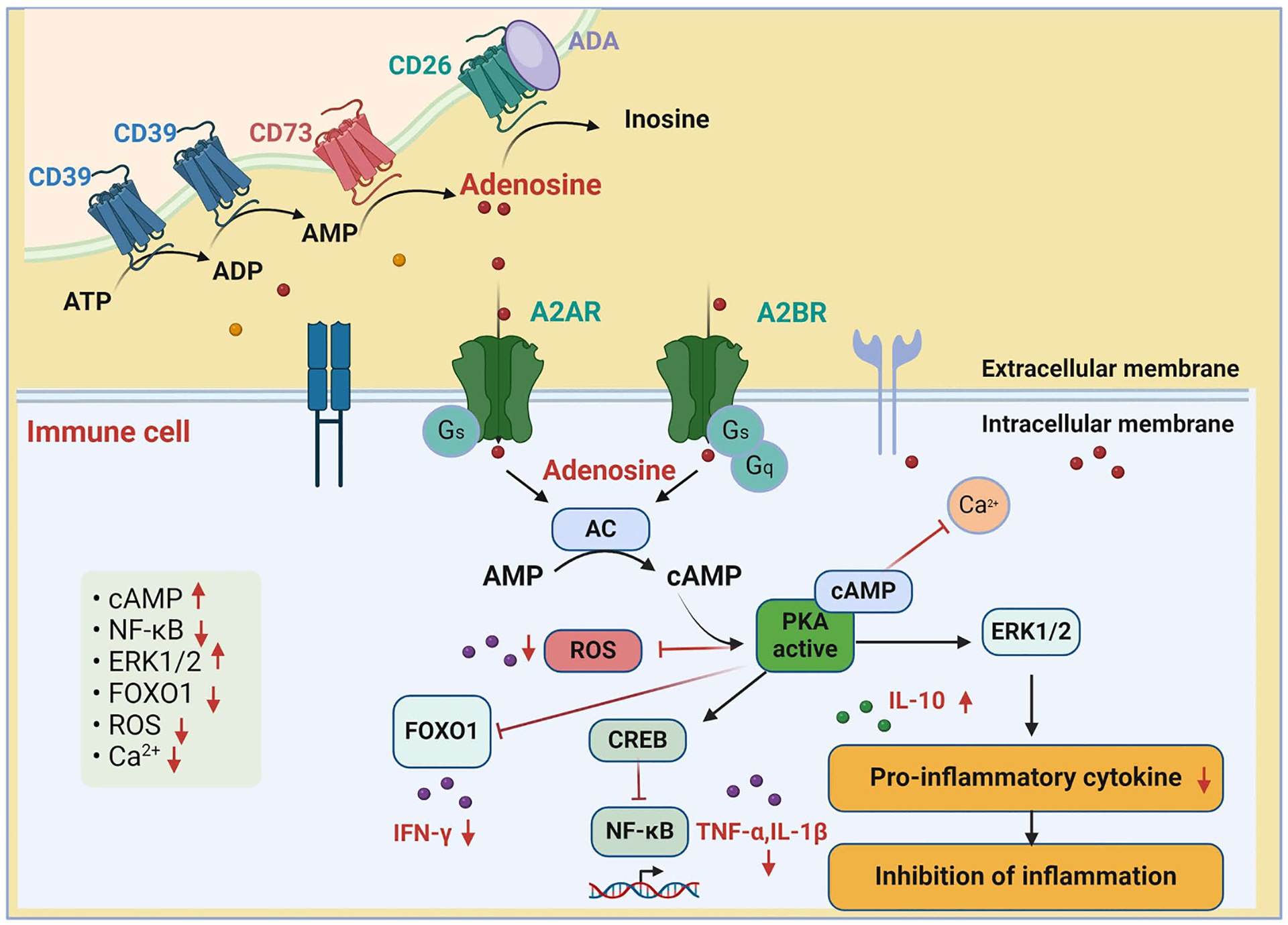
Schematic images of adenosine receptors in immune cells. The ectonucleotidases triphosphate diphophahydrolase-1 (CD39) metabolizes adenosine triphosphate (ATP) into adenosine monophosphate (AMP), and ecto-5′-nucleotidase (CD73) metabolizes AMP into adenosine. Adenosine is then converted to inosine by CD26-adenosine deaminase (ADA). The adenosine 2A receptor (A2A) and 2B receptor (A2B) are coupled to adenylyl cyclase (AC). AC converts AMP to cyclin AMP (cAMP), subsequently activating protein kinase A (PKA). Activated PKA leads to decreased reactive oxygen species (ROS) production, increased extracellular signal-regulated kinase 1/2 (ERK1/2) phosphorylation, reduced forkhead box O 1 (FOXO1) phosphorylation, decreased calcium secretion, and decreased nuclear factor kappa-light-chain-enhancer of activated B cells (NF-κB) activation. Ultimately, adenosine A2A and A2B receptor-mediated signaling inhibit inflammatory responses by suppressing the secretion of pro-inflammatory cytokines and enhancing the secretion of anti-inflammatory cytokines in immune cells such as T-cells and NK cells. TNF-α: tumor necrosis factor-α, IL-1β: interleukin 1β, IFN-γ: interferon gamma, IL-10: interleukin-10.

Neuroinflammation is a key factor in the development of metabolic syndromes [98,99], as well as various neurological disorders such as AD, PD, and multiple sclerosis (MS) [100]. In the CNS, astrocytes and microglia regulate the neuroimmune response and neuroinflammation by releasing inflammatory cytokines and reactive oxygen and nitrogen species [101,102]. A2AR plays an important role in mediating neuroinflammation and hippocampal neuronal loss [103], while also enhancing microglial function [104]. On the other hand, A1R expression in microglia regulates microglial activation and polarization [105]. Knockout mice lacking A1R have been observed to exhibit increased neuroinflammation and microglial hyperactivity [106]. A1R activation has been shown to decrease astrocyte proliferation and promote the production of nerve growth factor (NGF) [107]. Additionally, Ouyang et al. reported that adenosine signaling activation through A2AR acts as a critical regulator of inflammasome activation in macrophages [108]. A recent study demonstrated that the activation of A2AR could accelerate the production of pro-inflammatory cytokines such as interleukin 1β (IL-1β) through nuclear factor kappa-light-chain-enhancer of activated B cells (NF-kB) signaling and increase NLR family pyrin domain-containing 3 (NLRP3) activation in THP-1 macrophages [109]. Another study indicated that A2AR activation is involved in NLRP3 activation in microglia with increased glutamate levels [110]. Several in vivo and in vitro studies reported that A2AR antagonists can inhibit microglial activation and neuroinflammation [103,111,112], and A1R activation in microglia can reduce the production of pro-inflammatory cytokines [113,114]. A1R knockout mice were observed to exhibit increased activation of macrophages in the brain parenchyma [114]. Treatment with the A2AR antagonist ZM241385 was found to suppress microglial activation and inhibit pro-inflammatory cytokine secretion [115]. Furthermore, adenosine signaling modulates various immune cell functions [116–118] (Fig. 1). CD39 and CD73 expression in monocytes and macrophages induce the production of ATP and adenosine [118] (Fig. 1). Adenosine and adenosine receptors regulate the functions of certain lymphocytes such as T lymphocytes [118] (Fig. 1). Neutrophils produce ATP and adenosine by regulating CD39 and CD73 [119]. In endothelial cells, A2BR activation suppresses leukocyte adhesion and maintains leukocyte migration [120]. Additionally, A3R-mediated signaling activates ATP release and neutrophil migration [121]. A2AR can stimulate the activation of extracellular signal-regulated kinase 1/2 (ERK1/2) and inhibit NF-κB, leading to decreased interferon-gamma (IFN-γ) production and increased interleukin-10 (IL-10) in natural killer (NK) cells [122,123] and T-cells [124,125] (Fig. 1). The activation of A2AR can stimulate the activation of cyclooxygenase 2 (COX-2) and the release of prostaglandin E2 (PGE2), contributing to the inflammatory response [126,127]. Additionally, A2AR activation induces the release and synthesis of NGF [128], which can contribute to reduced neuroinflammation [129]. A3R activation in microglia induces the phosphorylation of ERK1/2 [130]. The reduced activation of A1R in macrophages can trigger the inflammatory response in the brain of patients with MS [131]. A2AR-mediated signaling has been reported to activate the anti-inflammatory response by modulating macrophages and neutrophil functions [132,133]. Adenosine and ATP can activate dendritic cells, which are antigen-presenting cells, and promote T-cell activation [118]. A2A antagonists and A1R agonists have been shown to modulate neuroinflammation and the immune system in the brain [134, 135]. Knockout of A2A in mice was found to increase the T cell-mediated inflammatory response in various tissues [136,137]. Moreover, A3R-mediated signaling was observed to induce anti-inflammatory effects in an autoimmune inflammatory disease model [138] and improve inflammatory cell infiltration in ischemic stroke models [139,140]. Treatment with IB-MECA, an A3R agonist, has been reported to reduce nitric oxide production and inhibit hippocampal neuronal cell death in ischemic stroke models [141,142]. A3R agonists can also inhibit NLRP3 activation [143], block the production of the pro-inflammatory cytokine IL-1β [143], and increase the secretion of the anti-inflammatory cytokine IL-10 [144]. The activation of A3R by the A3R agonist MRS5980 has been reported to enhance the secretion of anti-inflammatory cytokines in CD4^+^ T cells [145]. Overall, based on the literature, adenosine and adenosine receptors can regulate the immune and inflammatory responses by activating microglia, macrophages, and lymphocytes.

## 4. Adenosine signaling and BBB disruption

The BBB is composed of astrocytes, pericytes, and brain endothelial cells, serving as a crucial physiological barrier that separates the peripheral blood circulation from the CNS [146,147]. It selectively blocks potential neurotoxins, hydrophilic molecules, and metabolic products from entering the brain [146–148]. The BBB, also known as the neurovascular unit (NVU), plays a vital role in controlling barrier homeostasis and stability [149]. Metabolic syndromes such as diabetes can increase BBB permeability, leading to an increased risk of ischemic stroke [150]. Diabetes causes the thickening of the vascular capillary basement membrane and increases BBB disruption, resulting in neuroinflammation [151] and increased leukocyte invasion [152,153]. BBB breakdown in metabolic syndromes contributes to cognitive impairment [154–156]. Tight junction proteins such as occludin can anchor endothelial cells and maintain the structural integrity of the BBB [157]. A reduction in tight junction proteins has been observed in the brain of rodent models with obesity and diabetes [155]. A2AR expression in endothelial cells has been linked to BBB breakdown, leading to synaptic impairment and memory loss in diabetic mice with insulin resistance [158]. Astrocytes, the major constituent cells in the BBB, regulate BBB tightness and contribute to the maintenance of BBB integrity [159,160]. The four adenosine receptors are expressed in astrocytes, and astrocyte proliferation can be inhibited by A1R-mediated signaling [159]. Adenosine receptor signaling is known as a key mediator of BBB permeability [161]. A2AR activation induces NF-kB gene transcription [162] and downregulates the expression of the tight junction protein claudin-5 under inflammatory conditions [163]. The A2AR antagonist SCH58261 has been shown to cross the BBB [164] and maintain BBB integrity [165]. Agonists of A1R and A2AR have also been observed to contribute to tight junction protein integrity in cultured brain endothelial cells [32]. The CD39 and CD73 system modulates leukocyte migration stimulated by chemokines and immune cell adhesion to vascular endothelial cells [166]. In particular, CD73 is necessary for the entry of lymphocytes into the brain [167]. Adenosine receptors are expressed in brain endothelial cells, and the inhibition of CD73 or A2AR signaling suppresses the migration of leukocytes into the brain [167]. On the other hand, A2AR activation induces a significant increase in RhoA activity, leading to improved micro-attachment between endothelial cells and the extracellular matrix [168,169]. Based on the existing evidence, adenosine receptors play an essential role in BBB homeostasis by regulating tight junction proteins and maintaining the homeostasis of brain endothelial cells, astrocyte function, and leukocyte infiltration.

## 5. Adenosine, synaptic plasticity, and cognitive decline

**Fig. 2.**
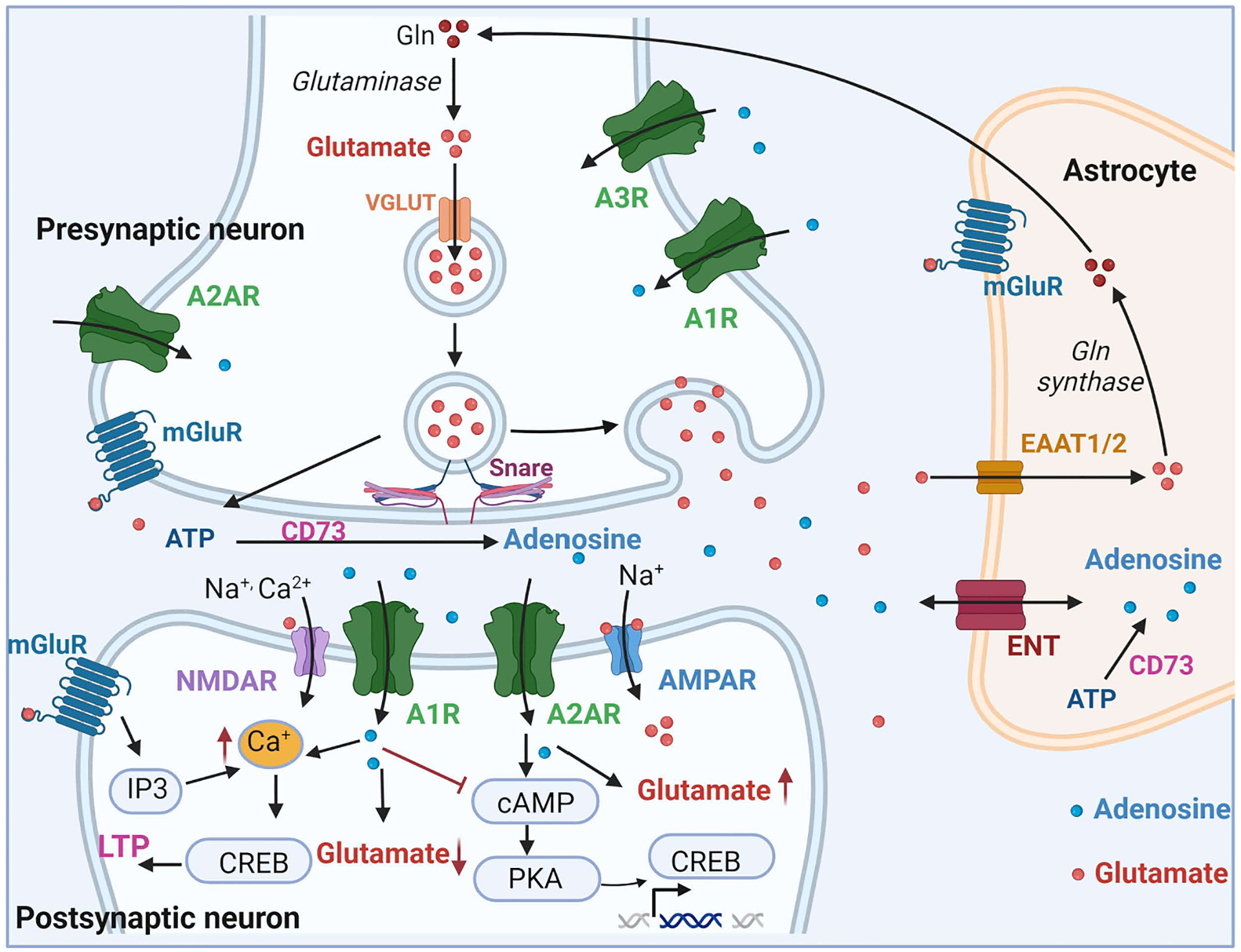
Schematic images of adenosine receptors in the synapse. Ecto-5′-nucleotidase (CD73) metabolizes adenosine triphosphate (ATP) into adenosine. The adenosine 1 receptor (A1R) inhibits the conversion of cyclin AMP (cAMP), thereby blocking protein kinase A (PKA)-cAMP response element-binding protein (CREB) signaling. AlR also suppresses the production of glutamate and calcium in post-synaptic neurons. The adenosine A2A receptor (A2AR) promotes cAMP-PKA-CREB signaling and enhances glutamate production in post-synaptic neurons. Equilibrative nucleoside transporter (ENT) mediates adenosine reuptake in astrocytes. LTP: long term potentiation, IP3: inositol-tri-phosphate; mGluR: metabotropic glutamate receptor of subtype, Gln: glutamine, NMDAR: *N*-methyl-D-aspartate receptor, AMPAR: α-amino-3-hydroxy-5-methyl-4-isoxazolepropionic acid receptor.

Metabolic syndromes are linked to the development of dementia and mild cognitive impairment due to synaptic failure [170,171]. An epidemiology study has shown that patients with diabetes have a high risk of dementia [172]. Similarly, patients with obesity often exhibit synaptic failure, memory loss, cortical atrophy, and an increased risk of dementia [173,174]. A2AR expression contributes to the production of pro-inflammatory cytokines [175] or the reduction of pro-inflammatory cytokines [176], affecting neuroinflammation and memory performance [177,178]. A study using an A2AR agonist (CGS 21680) reported that the activation of A2AR could modulate neuroinflammation by regulating inflammatory cytokine secretion dependent on glutamate concentration in a brain injury model [104]. Adenosine is involved in neuronal circuits, synaptic plasticity, and cognition [33]. A1R and A2AR are present in the dentate gyrus and CA1 hippocampal neurons, which are involved in cognition and memory consolidation [179]. A1R activation inhibits LTP, whereas A2AR enhances synaptic plasticity [33] (Fig. 2). A1R and A2AR work together to regulate the neural network and are located in excitatory synapses in the limbic cortex and cerebral cortex [19,180]. Specifically, A2AR-mediated signaling induces the secretion of glutamate [181] and *N*-methyl-D-aspartate (NMDA) receptor signaling in the hippocampus [182], which could increase synaptic plasticity [22,183] and synaptotoxicity [61] (Fig. 2). The inhibitory regulation of excitatory synaptic transmission is mostly attributed to presynaptic A1R in excitatory synapses [184]. The A1R-mediated inhibition of synaptic transmission is dependent on the ability of A1R to inhibit N-type calcium channels [185]. Presynaptic A2AR plays an important role in controlling the release of glutamate and blocking the A1R-mediated inhibition of synaptic transmission [186]. In addition, postsynaptic A2AR activation modulates NMDA receptors [187] and promotes LTP at excitatory synapses by facilitating AMPA receptor-mediated signaling and NMDA phosphorylation [188]. The overexpression of A2AR has been associated with spatial memory and behavioral impairment [175,189], whereas blockade of A2AR has been shown to prevent memory loss in animal models of AD [190–193]. In addition, A2AR-mediated signaling has been implicated in memory impairment in adult mice [189]. Neurogenesis plays a key role in enhancing learning and memory functions in neurodegenerative diseases [194]. Adenosine receptors such as A1R and A2BR are found in the subventricular zone, and treatment with A1R agonists can increase neural stem cell proliferation [195,196]. Adenosine signaling enhances brain-derived neurotropic factor (BDNF) signaling in the hippocampus, promoting neuronal cell survival and neural stem cell proliferation [197]. A2AR-mediated signaling can activate the transcription of the BDNF receptor TrkB, thereby inhibiting neuronal loss [198–202]. Some studies have suggested that A2AR-mediated signaling may contribute to memory deficits and the onset of AD [175,203–205]. The A2AR antagonist SCH58261 has been shown to prevent amyloid beta-induced synaptic dysfunction and memory loss [206]. Activation of A2AR in the hippocampal region can modulate the excitability of gamma-aminobutyric acid (GABA)ergic neurons [207], the production of GABA [208], and GABAergic synaptic stability [209]. A2AR is present in various brain regions including the olfactory tubercle, cerebral cortex, striatum, and hippocampus [210]. It is also expressed in astrocytes and neurons in the hippocampal region [211]. A2AR in astrocytes regulates neuronal excitability and cognitive performance [212,213]. A2AR antagonists have been shown to potentiate the release of glutamate [181] to AMPA receptors [84] and NMDA receptors [214,215], modulate astrocytic function [31,216], and reduce microglial activation [217]. In a mouse model of early AD with amyloid beta overaccumulation, A2AR has been implicated in synaptic plasticity and cognitive decline [218]. Additionally, the activation of A1R in astrocytes in the hippocampus suppresses fear memory formation [219], whereas the activation of A2AR contributes to contextual fear memory consolidation, resulting in fear generalization [220] and fear acquisition [221]. Furthermore, studies have reported that A1R activation inhibits excitatory synapses in the basolateral amygdala, whereas A2AR activation enhances inhibitory synaptic plasticity in the central amygdala [222], as well as the induction of LTP in excitatory synapses in the basolateral amygdala [221]. Adenosine cleaved from astrocyte-derived ATP induces the synaptic inhibition of pyramidal neurons via A1R activation mediated by somatostatin-expressing interneurons [223]. The activation of A1R leads to the inhibition of adenylate cyclase, resulting in a reduction in cAMP levels. This reduction, coupled with the blockage of voltage-gated Ca^2+^ channels, suppresses pyramidal neuron excitability [224,225]. In AD, the blockage of A2AR-mediated signaling has been shown to alleviate spatial memory decline by enhancing the synaptic plasticity of adult-born granule cells [226,227]. Elevated levels of A2AR have been reported in the hippocampus and cortex [31,228] of both AD patients [229] and an APP/PS1 transgenic AD mouse model [228]. The abnormal expression of A2AR may be linked to hippocampal neurogenesis in AD patients [229] and neural stem cell differentiation in the mouse brain hippocampus [230]. Studies have demonstrated that A2AR activation can improve neurogenesis and inhibit neuronal cell loss in a spinal cord injury mouse model [231,232]. A2AR knockout mice were observed to exhibit cognitive decline due to decreased neuronal cell proliferation and abnormal synaptic density protein expression in the hippocampus [233]. However, some studies reported that A2AR knockout mice did not exhibit cognitive impairment [193,206,234]. Treatment with the A2AR agonist has been shown to increase neurogenesis in the adult hippocampus and alleviate cognitive function impairment [235]. Conversely, the optogenetic activation of intracellular A2AR signaling in the hippocampus [236] and the overexpression of A2AR [192] have been found to decrease memory performance. Another study indicated that A1R and A2AR can form a complex with dopamine receptors such as D1 and D2 dopamine receptors [237]. The increased expression of A2AR may lead to dopaminergic neuronal cell death in the brain striatum [238]. A2AR interacts with the metabotropic glutamate receptor mGluR5 to form a heteromeric complex [239–241], and A2AR-mGluR5 interaction contributes to the enhanced release of neurotransmitters such as glutamate [242,243]. It has been shown that A2AR activation can inhibit glutamate uptake and the synaptic glutamate transporters GLAST and GLT-I [244]. In the AD brain of both young and old mice, the overexpression of A2AR in astrocytes has been associated with improved LTP [245]. A study has demonstrated that A2AR knockdown in hippocampal neurons can prevent fear memory retrieval in a traumatic brain injury model [246]. Furthermore, long-term caffeine intake, which non-selectively antagonizes A2AR, is known to attenuate fear memory formation in the amygdala [221,247]. The inactivation of A2AR in the striatum has been reported to enhance fear memory regulation [248] and alleviate spatial memory loss in a hypoxia mouse model [249]. A2AR-mediated signaling has also been linked to the decreased density of synaptic markers in the hippocampus, leading to memory deficits [205,250]. In addition, A3R may play a role in improving cognitive decline in traumatic brain injury [251]. Some studies have suggested that A3R could mediate the inhibitory effect of synaptic transmission in cortical neurons [252] and the facilitatory effect of glutamatergic neurotransmission [253,254]. The blockage of A3R has been shown to alleviate the impaired synaptic function of excitatory cortical neurons under ischemia conditions in vitro [255,256]. The A3R agonist 2-Cl-IB--MECA has been found to influence neuronal synaptic transmission and epileptiform activity in the rat hippocampal CA3 brain region [254].

Taken together, these findings indicate that adenosine receptors may play important roles in modulating synaptic plasticity, neurotransmitter secretion, spatial memory formation, and fear memory formation.

## 6. Conclusion

We review the existing findings on the role of adenosine receptor-mediated signaling in various neuropathological conditions associated with metabolic imbalances. First, adenosine receptor-mediated signaling enhances energy metabolism, brain insulin resistance, lipolysis, and lipid profiles under metabolic imbalance conditions. Second, adenosine receptor-mediated signaling ameliorates the inflammatory response by regulating the functions of microglia, macrophages, and immune responses through the modulation of lymphocyte migration and infiltration. Third, adenosine receptor-mediated signaling maintains BBB density and enhances the functions of astrocytes and brain endothelial cells. Lastly, adenosine receptor-mediated signaling improves synaptic plasticity and neurotrophic factor secretion, thereby facilitating the formation of spatial memory and fear memory. Overall, we highlight the therapeutic potential of adenosine signaling to address neuropathological issues associated with metabolic imbalances.

## Abbreviations:

BBB: blood-brain barrier
CNS: central nervous system
ATP: adenosine triphosphate
AMPA: α-amino-3-hydroxy-5-methyl-4-isoxazolepropionic acid
PKA: protein kinase A
LTP: long-term potentiation
LTD: long-term depression
ADK: adenosine kinase
ADA: adenosine deaminase
AMPK: AMP-activated protein kinase
TBI: traumatic brain injuries
PD: Parkinson’s disease
ALS: amyotrophic lateral sclerosis
AD: Alzheimer’s disease
ABCA1: adenosine 5-triphosphate-binding cassette transporter A1
ABCG1: adenosine 5-triphosphate-binding cassette transporter G1
ApoA1: apolipoprotein AI
HDL: high-density lipoprotein
LDL: low-density lipoprotein
NGF: nerve growth factor
IL-1β: interleukin 1β
NLRP3: NLR family pyrin domain-containing 3
NF-κB: nuclear factor kappa-light-chain-enhancer of activated B cells
COX-2: cyclooxygenase 2
PGE2: prostaglandin E2
ERK1/2: extracellular signal-regulated kinase 1/2
NVU: neurovascular unit
NMDA: N-methyl-D-aspartate
GABA: gamma-aminobutyric acid
